# Human in the collaborative loop: a strategy for integrating human activity recognition and non-invasive brain-machine interfaces to control collaborative robots

**DOI:** 10.3389/fnbot.2024.1383089

**Published:** 2024-09-24

**Authors:** Artur Pilacinski, Lukas Christ, Marius Boshoff, Ioannis Iossifidis, Patrick Adler, Michael Miro, Bernd Kuhlenkötter, Christian Klaes

**Affiliations:** ^1^Chair of Neurotechnology, Medical Faculty, Ruhr University Bochum, Bochum, Germany; ^2^Institute Product and Service Engineering, Ruhr University Bochum, Bochum, Germany; ^3^Chair Theoretical Computer Science and Artificial Intelligence, Ruhr West University of Applied Sciences, Mülheim an der Ruhr, Germany

**Keywords:** human-robot collaboration, brain-machine interfaces, human action recognition, sensor fusion, EEG

## Abstract

Human activity recognition (HAR) and brain-machine interface (BMI) are two emerging technologies that can enhance human-robot collaboration (HRC) in domains such as industry or healthcare. HAR uses sensors or cameras to capture and analyze the movements and actions of humans, while BMI uses human brain signals to decode action intentions. Both technologies face challenges impacting accuracy, reliability, and usability. In this article, we review the state-of-the-art techniques and methods for HAR and BMI and highlight their strengths and limitations. We then propose a hybrid framework that fuses HAR and BMI data, which can integrate the complementary information from the brain and body motion signals and improve the performance of human state decoding. We also discuss our hybrid method’s potential benefits and implications for HRC.

## 1 Introduction

Robots are becoming part of daily human life. From care homes to factories, robots help humans in repetitive, strenuous, or otherwise non-ergonomic tasks. They work independently or as human-controlled devices. For example, an assistive robotic arm can help a person in a wheelchair lift and grab objects that would otherwise be beyond their reach. However, humans are a collaborative species: working together is essential for many activities and humans excel at it. This is because humans can read the motor intentions of another human and immediately react to them (Blakemore and Decety, 2001). This is not possible for robots in this form, and human-robot collaboration is nowhere close to collaboration between humans (A positioning paper by the International Federation of Robotics, 2020). Unlike the presently available robots, the human brain comes equipped with computational machinery specialized for recognizing and predicting the intentions of other people (Blakemore and Decety, 2001). It can recognize a mistake within less than 100 ms (Spüler and Niethammer, 2015), making the brain’s cortical response, by design, a much faster signal source than any human motor action typically used in the context of correcting machine errors (such as pressing a button). In this article, we summarize the current state of the art in the field of collaborative robotics in terms of equipping robots with human activity recognition (HAR) systems and utilizing human abilities to initiate and recognize actions, particularly robot errors, in the brain-machine interface (BMI) framework.

A BMI is a device or system that enables direct communication between the brain and an external device, such as a computer or a robotic limb, by measuring and decoding the brain’s neural activity (Lebedev, 2014). The use of BMI to seamlessly integrate robotic equipment into human activity is, strictly speaking, not new (Ortiz et al., 2023). For example, the BMI framework for controlling a robot appropriately describes the interaction that occurs in the most recent neuroprotheses, where user brain signals are applied to control a robotic effector in reaching and grasping tasks (e.g., Aflalo et al., 2015) or walking (Quiles et al., 2023). While the fields of BMI and neural prosthetics are, by definition, using neural signals to control machine’s actions, to our knowledge, these methods have not been up to date used in dynamic human-robot collaboration. While modern neural prostheses are shared control systems integrating brain signals and autonomous control policies of the device there is, however, a substantial difference to cobots: neural prostheses are meant to be directly controlled by their user and are “embodied” (become part of the user’s motor repertoire), while cobots remain autonomous agents. Therefore, the difference between BMI in both approaches seems to be not in providing direct user control of the cobot (as in the case of neural prosthetics), but rather have the still autonomous cobot adapt to the user’s neural signals for action observation without the need for user cognitive effort or embodiment as in controlling neural prosthetics.

**Figure 1:**
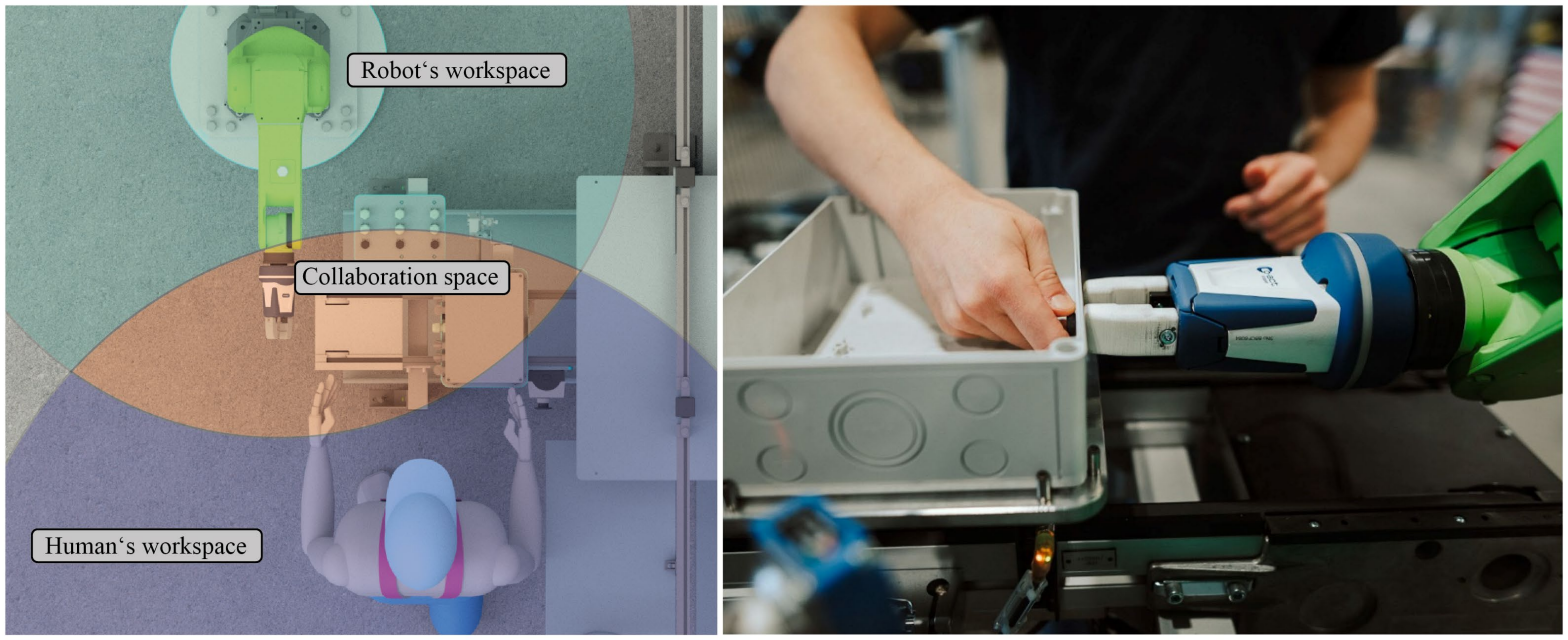
Left: definition of workspaces and the overlap as the collaboration space in HRC. Right: the interaction of human and robot when screwing together a switch box for photovoltaic systems. Here, the robot holds the screw while the human screws the nut.

Regular industrial robots perform pre-programmed actions in specifically prepared, highly structured environments. They have therefore little need for perception and no on-the-task interaction with human users. By contrast, collaborative robots are expected to share an environment with a human operator as can be seen in Figure 1, and therefore must detect the presence of a human operator or at least need to sense any form of collision and initiate a stop.

In HRC applications, the workspace is divided into the robot’s workspace, the human workspace, and the interaction space, in which both the robot and human workspace overlap (Figure 1). The planning of robot actions or movement paths strongly depends on the robot’s sensory input, as the robot works closely with a human in a dynamic environment and unplanned changes can therefore occur in the production process. These dynamic changes must always be recognized to eliminate the possibility of collisions with the environment or humans in the worst case and can lead to path replanning, force limitation, or speed adaption of the robot (Miro et al., 2022). The sensors used to secure the operator’s safety, based on ISO TS 15066, either detect the intrusion of people into the workspace or detect a collision with an operator.

For many interactive working scenarios, the classical human-computer interfaces, like the keyboard, buttons, or mouse, interfere with the user’s own manipulation tasks as they may require the user’s hand to leave the assembly workspace to type or control a pointing device. The need for a natural and intuitive user interface is thus obvious. Such an interface may be based on the robot’s recognition of user actions. For instance, the user may specify an object by naming it, exploiting object recognition capacities of the system, or by pointing at it, using gesture recognition capacities of the system. Due to the stringent safety requirements for HRC applications, it is crucial to assess whether BMI meet the necessary safety standards for use in HRC. In this context, the recognition and interpretability of human actions by technical aids are examined in detail. Subsequently, actions that have been inadequately detected are explored, along with the possibilities for improvement, their current technical status, and the challenges involved in implementation.

## 2 Human action recognition

Currently, various safety systems are employed in HRC, primarily detecting human entry into the working space (e.g., laser scanners) or collisions with the robot (torque sensors). Although these established systems ascertain the safety of the human worker, they do so only after the executed movement and do not distinguish between different body parts. One of the challenges lies in the fact that movements of different body parts are mostly part of complex movements of various body parts without the possibility of capturing thoughts or intentions (Aggarwal and Ryoo, 2011; Jegham et al., 2020). Basically, according to those authors, human body activities are subdivided into four categories depending on the involved body parts and their complexity (Aggarwal and Ryoo, 2011; Jegham et al., 2020):

- Gesture: Non-verbal communication involves conveying specific messages through visible bodily actions, excluding verbal or vocal elements, and can include hand, facial, or other body movements.
- Actions: It constitutes a series of physical movements performed by an individual, encompassing activities such as walking and running.
- Interactions: It involves a series of actions performed by a maximum of two entities, with at least one participant being a person and the other potentially being either a human or an object.

Group activities: It encompasses a mixture of gestures, actions, or interactions, involving a minimum of two performers and one or more interactive objects. For collaborative robotics, however, the focus is primarily on the interaction with robots and, above all, actions. For the purpose of this review, we will focus on a specific class of actions, that is overt bodily movements, Actions and interactions are closely related and cannot always be clearly distinguished from each other without using a system that enables HAR. In the subsequent step after the HAR, a human pose estimation is to be derived based on the detected data, with which a relation to the robot and its movement can be calculated.

**Figure 2:**
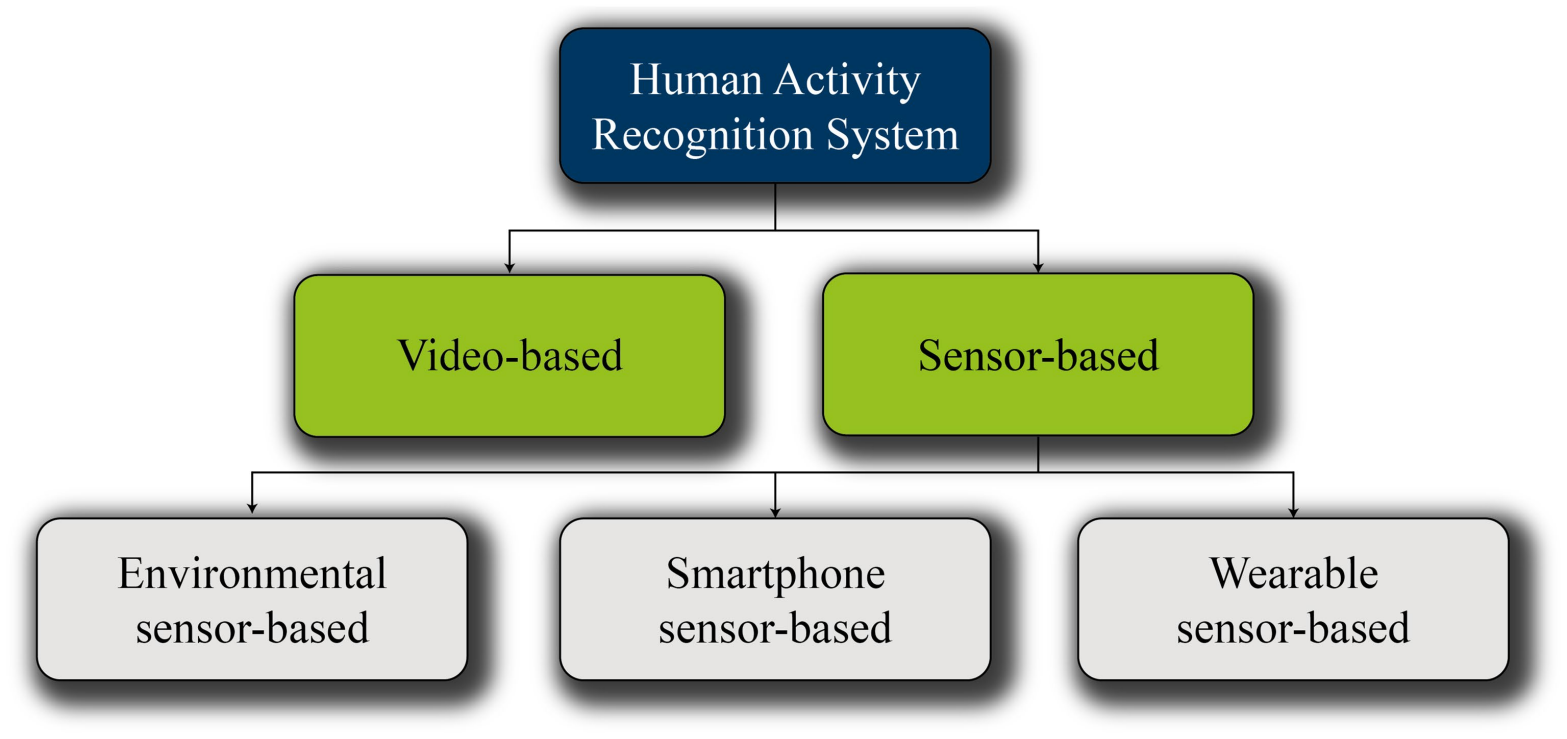
Approaches for HAR systems (Antar et al., 2019).

There are various HAR systems, divided into video-based and sensor-based systems, see Figure 2. Video-based systems use high speed cameras for precise tracking of body parts (such as hands). The sensor-based system, in turn, is subdivided into environmental, wearable, and smartphone-based (Antar et al., 2019). All these recognition systems are only reactive and capture the action after or during the execution of the movement. This contrasts with the use of BMI for EEG measurement, discussed in this context as a means of detecting and predicting human movements. In this case neural data from specific brain areas signal the movement before its actual execution (Planelles et al., 2014). This results in a significant time advantage even in the pure detection of a movement, as it enables a proactive measure to be taken before the person physically performs the movement.

In addition to the disadvantage of purely reactive motion detection, established systems such as video-based HAR face further challenges in this context. Anthropometric variation is a challenge since each person has, i.e., their own body size proportion or flexibility, which results in high variability of possible movements and intentions to be detected or predicted. Other optical effects like multi-view variation, cluttered and dynamic variation, or occlusion cause difficulties in the detection task (Jegham et al., 2020). Contamination, such as dust or sensitivity to changing light conditions in the industry, also reduces the reliability of such systems, which is particularly important in safety-relevant contexts such as HRC (Halme et al., 2018). This is why HAR systems may be insufficient for efficient responsive HRC, leading researchers to search for other sources of estimating the human state. Nevertheless, it should be mentioned that actual challenges are also being addressed in the field of HAR using various approaches (including AI-based approaches) and that research is also being conducted in the area of human action prediction (Kong and Fu, 2022).

In recent decades more attention has been put on the inclusion of BMI in robot control loops. The current range of functions and reliability of the sensor-based approaches, like wearable or smartphone-based, are unsuitable for use in the HRC context alone. However, the three standard wearable sensors, such as accelerometer, magnetometer and gyroscope (Minh Dang et al., 2020) can be used for HAR and collect relevant information, which can supplement a HRC safety system. Wearable sensors for detecting eye movement, heart rate, respiratory rate, and body temperature, for example, can also provide relevant information and improve the prediction as part of an overall system. Nevertheless, the BMI has a higher potential for use in HRC applications because, in addition to the movement detection before execution, it also has the potential to improve human pose estimation with qualitative information. Simultaneously the BMI avoids all optical problems because it uses a completely different measurement principle. A BMI has the potential to predict the movement up to half a second before it is executed (Planelles et al., 2014). This enables the robot to stop even before a movement is executed, which leads to a collision. As a result, the collision forces between humans and robots would be significantly reduced, which would enable higher robot operating speeds.

## 3 Brain-machine interfaces

Brain-machine interfaces can be used for various purposes, such as researching, mapping, assisting, augmenting, or restoring human cognitive or sensory-motor functions. BMIs can be classified into invasive and non-invasive. Invasive systems require the implantation of electrodes which are either connected to peripheral efferent and afferent nerves (Lebedev, 2014), or placed on top of the brain tissue or penetrating it. Depending on the usage, typically, the motor cortex (Hochberg et al., 2006) or other motor-related areas like the parietal cortex (Aflalo et al., 2015) are targeted. Non-invasive BMIs utilize signals that can be obtained without surgery, for example, electroencephalography (EEG) or functional near-infrared spectroscopy (fNIRS). As invasive BMIs are usually clinical testing devices and are not possible to use with healthy humans, in this paper, we focus on non-invasive EEG-based BMI, which record neural activity through electrodes placed on top of the scalp. For BMI purposes, a frequent method to train the system is to use known EEG signatures of action execution error (Chavarriaga et al., 2014; Fidêncio et al., 2022) or sensorimotor rhythms (Yuan and He, 2014).

### 3.1 Error signatures in EEG

Event-related potentials (ERPs) are changes in the brain’s electrical activity that occur in response to specific stimuli or events. ERPs are measured by recording the brain signals using EEG and then averaging the signals across many trials to isolate the brain response from the background noise. Error-related brain activity or error-related potential (ErrP’s) is a specific type of event-related potential elicited after subjects committed errors in a given task (Spüler and Niethammer, 2015; Fidêncio et al., 2022). This brain activity pattern is characterized by a potential deflection 50–100 ms after a subject’s erroneous response, termed the error-related negativity (ERN), appearing over the fronto-central scalp areas as a negative potential (Ne), following a subsequent centro-parietal positive potential (Pe) (Fidêncio et al., 2022). A similar medial-frontal EEG pattern has been reported to appear after the presentation of “feedback,” i.e., the delayed result of a choice or action. This feedback-related negativity (FRN) is between 200 and 300 ms after feedback onset (Fidêncio et al., 2022).

In the frequency domain of EEG signals, several studies demonstrated frequency-specific power modulations in response to erroneous action execution in different tasks. Effects were mainly found in lower frequency bands, such as delta (1.5–3.5 Hz), theta (4–7 Hz) (Kolev et al., 2005; Yordanova et al., 2004; Moreau et al., 2018), alpha (10–14 Hz) (Carp and Compton, 2009), and beta (15–30 Hz) bands (Vidal et al., 2003). Welke et al. (2017) also showed error-related modulation in frequency ranges higher than 30 Hz, including a high-gamma range (50–150 Hz). Koelewijn et al. (2008) and Moreau et al. (2018) demonstrated an effect of the correctness of observed actions on beta and theta power modulation. More importantly, however, Moreau et al. (2018), showed that theta error modulation was visible during joint actions requiring observation and monitoring of both own and other agent’s actions. Such suggested spatial separation between channels conveying own vs. observed error signals can greatly improve the chances of successfully decoding signals related to cobot errors in joint actions.

### 3.2 Sensorimotor EEG activity

EEG signals frequently used for BMI are related to sensorimotor functions, such as movement planning or motor imagery, expressed in time or frequency domains. The ERP used to predict movement is readiness potential (RP), described as a buildup of EEG activity preceding movement onset (Schurger et al., 2021). The use cases of RP for BMI are limited due to its unspecificity with only few notable exceptions like controlling a lower limb exoskeleton (Jeong et al., 2017).

Another class of EEG signals used in BMI are sensorimotor oscillations/rhythms (Yuan and He, 2014). One of the basic rhythms are mu waves (7–15 Hz) which indicate a switch between resting and active states of the cortical motor areas (Pineda, 2005). A common application is to use mu rhythm desynchronization to detect movement intention which, like in the case of RP, can be achieved before the movement starts (Yuan and He, 2014; Lazarou et al., 2018). This way, movement can be predictively decoded from sensorimotor EEG activity, as opposed to ErrP’s which respond to the effects of an already executed movement.

## 4 Use of brain signals for controlling robots

Brain signals have been used to control collaborative robots in several use cases mainly focusing on robot action observation. The selected papers we refer to below demonstrate different approaches to the inclusion of non-invasive BMI in controlling robots, from passive observation to predictive monitoring of collaborative action performance.

As an example of using BMI for controlling observed actions, Welke et al. (2017) designed a task in which participants watched a robot arm pour liquid into a cup. The robot performed the action either erroneously or correctly, which means it either spilled the liquid or not. In another task, their participants observed two different types of robots, humanoid and non-humanoid, grabbing a ball. The robots either managed to grab the ball or it would make an error. The number of trials per condition was 360 in Exp 1 and 200 in Exp 2. In both experiments, the authors used a real-time pipeline for decoding the multivariate EEG signal (128 channels Waveguard gel cap (ANT Neuro)) and successfully attempted to decode errors and robot type using the frequency spectrum of brain oscillations from 1 Hz to 144 Hz. The authors show that decoding accuracies for decoding both error detection and robot anthropomorphism were higher at lower (< 20 Hz) frequency bands. These results indicate that the brain responses not only encode the robotic partner’s errors but are also additionally modulated by the robot’s anthropomorphism, further indicating that the brain differentiates in action observation of humanoid vs. non-humanoid agents.

Salazar-Gomez et al. (2017) EEG-measured error-related potentials to specifically design a closed-loop BMI for robotic control. ErrP signals were decoded from a human operator in real-time to control a robot during a discrete object selection task. Each of the 5 closed-loop BMI blocks contained 50 trials and lasted 9 min. The EEG system was 48-channel g.USBAMP (g.Tec). The observers watched the robot perform object-grasping tasks while their EEG signals were recorded to capture ErrP. In the case of ErrP detection, the robot action was corrected to choose the correct target. In some trials, the controller randomly decided not to inform the robot of an error to induce a secondary ErrP in the subjects. The use of ErrP was successful in online correction of robot errors. Moreover, in offline analyses, the authors also discovered the existence of a secondary ErrP when the human observes that the robot has incorrectly interpreted its feedback. This signal was described as easier to classify than the original error, and the authors suggest its usefulness in improving the performance accuracy in a closed-loop BMI scenario.

To better study the cognitive state of users in joint actions, Singh et al., 2020 described a somewhat different class of ErrP, arising due to a conflict in predictions and action outcomes. They call this signal prediction error negativity (PEN). Unlike in most other studies, the authors used an active HRC paradigm in which the user and collaborative robot could encounter an obstacle. Their results show higher PEN for cognitive conflict conditions compared to normal conditions and a statistically significant difference between different levels of PEN. These results indicate that cognitive conflict can be detected in dynamic HRC settings and be read as another type of signal, thus improving the detection of ErrPs and user state classification.

The major weakness of ErrPs is that they are evoked after the robot has committed an error, which might be costly or dangerous in dynamic contexts, such as assembly lines or autonomous driving. To address these limitations, Wang et al., 2022 proposed a novel BMI system where robot intentions were communicated before actions and the operator continuously evaluated these robot’s intentions. This way ErrP were detected before the robot would commit an error (that is, predicting the possible error). They tested their BMI framework via an experiment where a robot performed a target-reaching task. The high classification accuracy (77.57%) for predictive ErrP’s demonstrated that the predictive ErrP-based BMI was feasible for human–robot intention and has the potential to broaden the range of applications for ErrP-based BMIs. These above results show that the utility of neural signals for controlling robotic actions has been demonstrated mainly for passive observation. The use of BMI for responsive cobot adaptation would need to take one step further: towards the use of BMI for improving cooperation in situations where both humans and robots are simultaneously in motion. This, however, would require facing several technical challenges related to physiological data acquisition and processing.

## 5 Challenges for the use of BMI in HRC

Although BMI can be investigated and used not only in the context of the safety of robot applications and HRC, their use as a complementary safety system still faces substantial challenges. In addition to HRC-specific challenges, there are also cross-technology and cross-context challenges when using BMI. These include, for example, challenges in the acceptance of wearing an (Rashid et al., 2020) EEG cap, the need for intensive individual training, different physical head geometries and challenges in the transferability of results and evaluation algorithms due to the high individuality of brain currents with simultaneous high demands on accuracy and reliability. There are also risk factors associated with the use of BMI and HRC, particularly in terms of safety, which preclude their direct use for accurately detecting current and future human movements. BMI alone may identify movements with a high degree of uncertainty, posing a risk of resulting in different actual movements. Incorrect recognition of the arm’s direction of movement, inaccurate estimation of movement speed, or registering a movement of the wrong body part can significantly endanger the safety of individuals. To assess safety risks, it is essential to closely examine current challenges and evaluate their effects and interactions within the overall process.

### 5.1 Extending current regulation standards

The ISO TS 15066 safety standard is a cornerstone in ensuring the safety of collaborative robots. This standard delineates technical safety requirements and specifications for the working environment, providing clear guidelines for HRC. These guidelines encompass crucial factors like maximum robot movement speeds and acceptable limits for collision forces on various body regions. ISO TS 15066 extends the existing ISO 10218, which lays down comprehensive safety requirements for industrial robot systems. Specifically, ISO TS 15066 outlines protocols for managing hazards, evaluating associated risks, and implementing measures to eliminate them. This standard is a pivotal framework for ensuring collaborative robot’s safe integration and operation within diverse working environments. When using BMIs as a part of a HRC safety system, these requirements must also be met in connection with HRC, and potential sources of errors such as motion artifacts, signal unspecificities, and decoding latencies must be recognized or avoided with sufficient reliability.

For the use of BMIs in HRC scenarios, there are no specific regulations or guidelines regarding the minimum latencies for signal decoding, additional risks, and minimum safe spaces. The compilation of initial guidelines for secure implementation in accordance with regulations must, therefore, be consistently considered and treated separately as a socio-technical component during the development of the interaction option. In this context, also the consideration of acceptance by operators is always a crucial factor for a successful implementation and adaptation of new technologies (Linsinger et al., 2018). That must be considered when introducing BMIs in HRC. However, addressing and understanding operator acceptance is likely to be an interesting next step in future development.

### 5.2 Determination of the collision areas and the robot’s trajectory

Certainly, besides safety considerations, productivity is the main aspect that needs to be taken into account for any robotic application in a production context. Productivity, here defined as a measure of the most time-efficient path of executing movements in robot-based applications, represents the underlying optimization problem. The goal is to minimize the time it takes for the robot to complete its task, allowing for the shortest possible path executed at the highest feasible movement speed, all while not disrupting the employee’s workflow. Balancing safety and productivity are key to an effective and efficient HRC.

Solving the general problem of determining the resulting movement of the robot in terms of maximum forces, movement speed, and planned path based on measured uncertainties for a human action is essential. The maximum speed and forces depend on the human body regions potentially affected in the event of a collision with the robot. Moreover, the mass carried by the robot is relevant for determining the collision force, as a higher carried load additionally lowers the robot’s movement speed. Adapting the robot’s actions involves determining possible human movements and coupling them to the robot’s movement commands. For a deterministic collaboration scenario, where the human actions are known and certain, this has been done and can be found in Glogowski et al. (2021). In this regard, it is crucial to investigate the relationship between the probability of an action occurring, its impact on the size of assumed collision spaces between body limbs and the robot shell, and the resulting trajectory of the robot. The complexity of this task depends on various factors, including the available sensors, the precision of measurements, the dynamics of the robotic system, and the desired accuracy of trajectory adjustment or recalculation. In essence, it can be considered relatively straightforward when clear sensor information is available and the robot is well-modeled, or complex when uncertainties or intricate dynamics need to be taken into account.

### 5.3 Motion artifacts

Any mobile setup in the experiment is always known to introduce a high number of artifacts in EEG data due to movement. While gel-based electrode systems in EEG are more resistant to such motion artifacts, popular BMI solutions base on dry electrodes because of their easiness of use, such as that they do not require long setup times (McFarland and Wolpaw, 2017; Kam et al., 2019). Motion-related artifacts can be also dealt with on data processing stage by using established artifact-removal approaches in EEG data processing (such as channel-based template regression procedure and spatial filtering, independent component analysis, etc.) (Jamil et al., 2021). Alternatively, machine learning can be used to mark EEG segments affected by motion-related artifacts (Salehzadeh et al., 2020). Newer methods for recording brain data in moving humans, such as MOBI (Gramann et al., 2011), can be adopted to get a higher signal-to-noise ratio. In any case, an array of methods can help ensure that motion-related artifacts do not influence the resultant EEG signals.

### 5.4 Combining EEG with other physiological signals

While here we consider HAR only in relation to overt body movements, several methods for measuring and using real-time human activity have been proposed for using in different HRI frameworks. EEG signals for error might not just be difficult to detect because of signal noise but also because of cognitive interference. The dissociating of signal sources (i.e., error vs. cognitive inferences oculomotor/hand programming etc.) is a standard issue in BMI research (e.g., Hajcak et al., 2005) and there are several ways of disentangling the key signals (such as ErrP) through experimental designs (see, e.g., Chavarriaga et al., 2014). In addition, one may consider further potential mitigation measures such as validating error detection in EEG by combining with other signal sources (e.g., pupil metrics and biomarkers). For example, pupil responses have been demonstrated to reflect error perception reliably (Maier et al., 2019). Similarly, pupil diameter changes signify, e.g., cognitive focus and as such can be used for adaptive control of robots as proposed, e.g., by St-Onge et al. (2019). Other eye movements can be utilized to indicate the user’s current locus of visual attention (Krol et al., 2017). Other wearable sensors such as pulse or galvanic skin response meters were successfully utilized to measure users’ stress and trust level and provide real-time information about user state (Leeb et al., 2011; Hald et al., 2020). As all these signals alone might lack specificity in detecting user state (such as boredom, stress, etc.), hybrid methods have been proposed fusing between different signal types. These methods have been demonstrated, e.g., for combining eyetracking and EEG (Krol et al., 2017; Vourvopoulos et al., 2019). Moreover, integration of electromyographic (EMG) activity has been shown to result in a better and more stable BMI performance in motor tasks (Leeb et al., 2011). Using further sensors in fusion with EEG data can increase error detection substantially, especially when combined with double-step decoding as an additional validation method. Such fusion has been successfully tested showing improved detection of EEG events when measured together with eye signals (Vourvopoulos et al., 2019). When combined with machine learning, this sensor fusion will also be faster, allowing the detection of faster physiological signals and the prediction of possible errors that are anticipated to occur based on prior decisions during the HRC. In addition, fusion allows a cross-modal reconstruction module to learn dependencies between simultaneously recorded data streams and based on that reconstruct signal data in case one modality is missing. Compared to uni-modal classification, this has been demonstrated to provide a more robust output by the decoder (Vourvopoulos et al., 2019; Rahate et al., 2022).

To warrant more decoding accuracy analysis, BMI models can be trained to decode well-described error-related signals such as ERN, ErrP, or error modulation of frequency bands. Since the ERN is a fast and robust brain signal, its detection can be improved using methods effective at filtering out the noise such as a double ErrP checking algorithm (Cruz et al., 2018). A similar approach was tested successfully in the context of passive observation of cobot actions, increasing ErrP decode performance to about 90% (Salazar-Gomez et al., 2017). Although this method is slower than single-step decoding, it helps improve decoding accuracy. Additionally, the use of machine learning methods (operating over spectrograms or time series of the EEG signals) can help detect novel neural markers of cobot error and their related EEG channels that have not been described in the previous literature.

### 5.5 Decoding latencies

Giving the operator’s brain the responsibility of discovering errors faces an additional risk regarding the potential impact (in a critical system) of the combined latency of (a) signal production by the worker, (b) signal detection by the device, (c) validation of the signal (is the operator really producing an error signal), and (d) acting on the error signal. Previous experiments show that the accuracy of single trial detection can range between 65 and 95% (depending on the task and the participant). For this reason, reliable ErrP detection can be achieved using methods that have an increased correct detection rate such as double filtering (see, e.g., Cruz et al., 2018). However, more importantly, error monitoring in a more holistic way, i.e., considering the information from other biosignals (e.g., electromyography) and behavior (e.g., hand velocities, pupil reaction) can improve detection accuracy and speed. The detection of ‘complete’ ErrPs can be achieved in a time window of around 500 ms after error. Pupil reactions, for example, can be detected faster (Bergamin and Kardon, 2003) allowing the system to make classification decisions in a timely manner. Furthermore, one can focus on reliably capturing the fast ERN by validating it by ‘whole’ ErrP detection to speed up the system, compensating at least for issues (b) and (c) as listed above. Furthermore, one can be not only interested in an immediate reaction of the robot based on error recognition but also in accumulating error information (that can be associated with error likelihood) to make the cobot learn/adapt its behavior through reinforcement learning, which will increase the evidence of error in certain repetitive wrong cobot’s actions. This will further compensate for (a) and (d).

An interesting alternative seems to be provided by error prediction models in which robot intentions are communicated before actions and evaluated predictively and, therefore, can be detected and errors communicated back to the robot earlier than in feedback-based ErrP approaches (Wang et al., 2022).

## 6 Extending the BMI functionality with HAR for use in HRC

BMI and HAR are two techniques that can be used to measure and analyze the brain and body signals of a human. The multiple limitations of either of these methods alone make them a limited source of control in a critical system due to the risk to human operators. A promising way to address this challenge is to use hybrid approaches that combine EEG and HAR systems (Hussain et al., 2023). While sensor fusion is not novel, as described above, the addition of HAR can provide substantially more precise classification of the human state. This is a substantially novel concept and has seen limited practical demonstrations and theoretical descriptions of the framework to date, with a few notable exceptions such as Hussain et al. (2023). One possible way is to use a model that integrates the data from both modalities and applies machine learning algorithms to decode the human state, such as attention, emotion, intention, etc. as HAR algorithms are capable of Gupta et al. (2022). Such a hybrid system can leverage the complementary information from both modalities and achieve a more accurate and robust decoding of the human state in HRC. For example, human motion intention can be detected from BMI before the motion starts, thereby improving HAR. On the other hand, HAR can help provide contextual cues, reduce artifacts, and enhance feature detection in BMI. There are several conceivable use cases where BMI performance can be improved thanks to HAR:

- Using HAR to recognize the gestures and actions of humans and using them as labels or priors for the EEG classification or reconstruction (Salehzadeh et al., 2020). In HRC this can be, for example, detection of user performing a corrective movement or adapting their own movement in response to robot action. These detected corrections can serve to improve detection of ErrPs in data.
- Using HAR to extract features of human motion and fusing them with the EEG features to improve the representation and discrimination of the human state such as tiredness, boredom, etc. (Hussain et al., 2023).
- Using HAR to model the social and emotional aspects of the human state, such as gaze, facial expression, and speech, and incorporating them into the EEG decoding.
- As predictive communication of robot intentions has been presented to improve ErrP detection, the same can be implemented using HAR and predictive decoding of human actions, such as predictively intercepting human hand target based on motion kinematics and trajectory.

### 6.1 Concept of a hybrid approach in HRC

**Figure 3:**
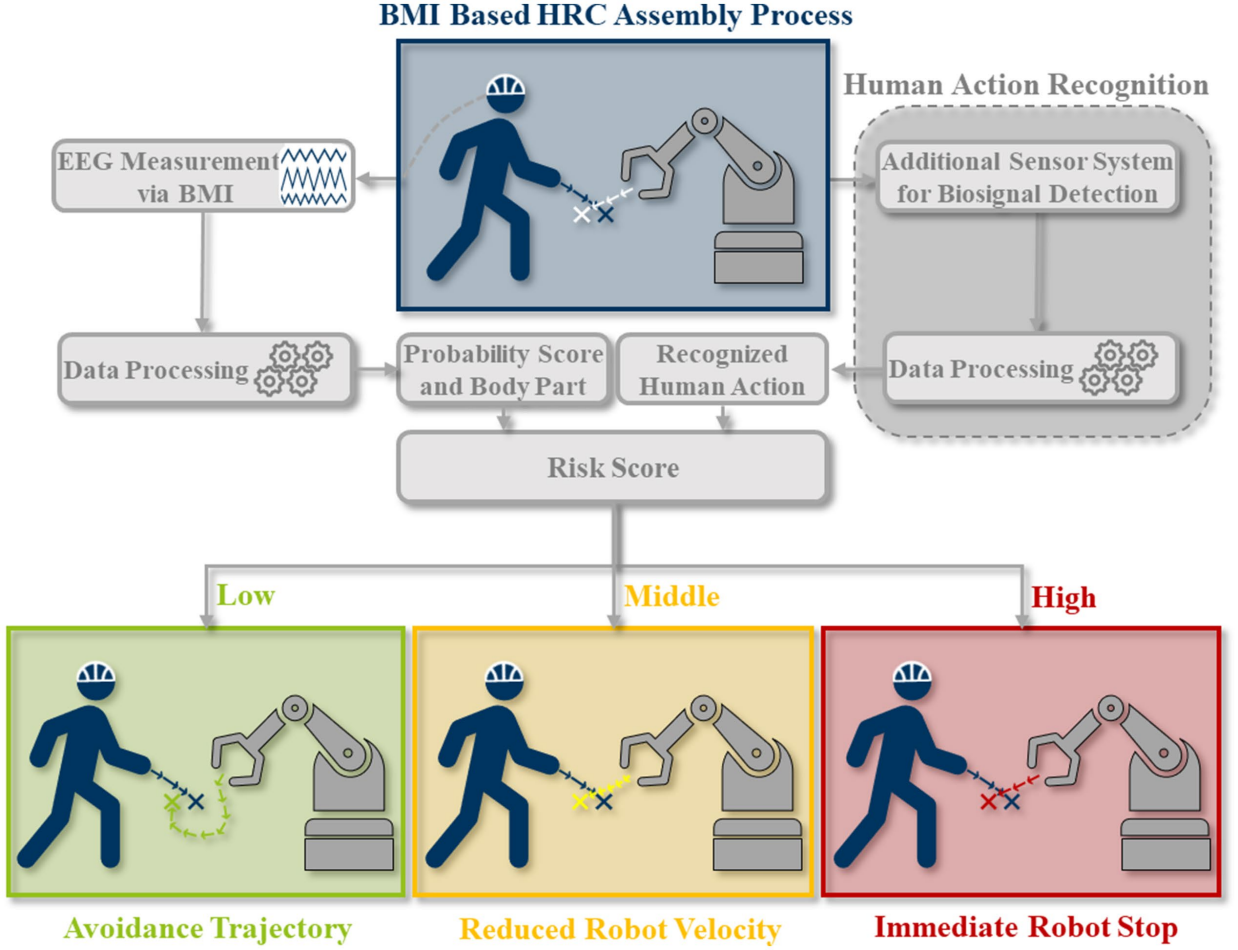
Concept of an optimized HRC system with the combination of BMI and HAR integration for a theoretical collision between the human and the robot.

A fusion model using BMI and HAR as input sources can be used in the context of HRC-based assembly processes and, thanks to the hybrid approach, serves as an optimized sensor system for HRC. The proposed model is not limited to using wearable sensor or video-based motion tracking but assumed to include several sensors providing comprehensive estimate of human activity, fusing motion kinematics, eye tracking, and other sensors as permitted and feasible for a given use case. This allows setup versatility and context-oriented signal classification, improving HAR. The BMI and the sensors for the HAR independently record measured values of the human operator in the assembly situation, which are processed and calculated into a risk score in the next step. Based on the risk score, a case differentiation is made for a theoretical collision between humans and robots. Depending on the clarity of the measured values and the safety risk for the human in the specific situation based on the applicable and described guidelines, the robot controller is instructed to either calculate and approach an avoidance trajectory, reduce the speed to a safe value in accordance with the HRC guidelines or initiate an immediate robot stop. This concept is illustrated in Figure 3. Based on Glogowski et al. (2021), these possible scenarios and robot reactions can be categorized and described as follows:

1. Risk score *low*: In the scenario of a high probability of human movement occurrence and low movement speed, the location of a human limb can be anticipated with confidence. Consequently, the robot’s path can be replanned, and a collision-free trajectory is ensured.
2. Risk score *middle*: In cases of high probability of occurrence and high movement speed, the robot trajectory is still planned to be evasive, but the speed is initially reduced. As the movement speed of the human exceeds that of the robot, the collision volumes must be increased so that sufficient braking time is available in the case of a collision. Ensuring the safety of the human is crucial in these scenarios, especially when there is an overlap in the movement speeds of the human and the robot.
3. Risk score *high*: In instances of high or low probability of human movement occurrence, and ambiguity of the measured value evaluation, thus an inability to detect the human movement speed and path occurs, a safety stop of the robot must be initiated.

The most important factor influencing the calculation of the risk score and the safety of the human is the behavior of the human itself. The outcomes in detail depend on both the probability of human movement occurrence and the speed at which it happens. This results in so-called collision volumes. They are calculated as buffer zones for the theoretical case of a collision to ensure the safety of human, their size depends on the human’s and the robot’s movement velocity (Corrales et al., 2011). As mentioned earlier, numerous factors, such as artifacts, influence the probability of occurrence, essentially representing the measurement uncertainty of the BMI. The robot’s reaction is also contingent on the speed of human movement. For example, adaptive evasion becomes infeasible if the movement speed surpasses the robot’s movement speed, necessitating a safety stop. Conversely, if the human’s movement speed is lower than that of the robot, the robot can devise and execute an evasive trajectory. Both the probability of occurrence and the speed of movement impact the assumed collision volumes positioned along the human skeleton for collision calculations. As the probability of occurrence decreases and human movement speed increases, the collision volumes expand to accommodate the larger potential area of the human’s actual position.

### 6.2 Potential of a hybrid approach in practical HRC applications

Regarding maximum movement speeds, the standard for HRC imposes relatively low limits compared to standalone industrial robots, which move at several meters per second. In most cases, a maximum moving speed of 250 mm/s is specified, which may only be exceeded under the condition that the working areas of the robot and human do not overlap to avoid human injury, as described by Vysocky and Novak (2016). Adherence to this specification is crucial to maintaining a safe working environment during interactive robot operations. This remains a significant drawback of HRC since a low movement speed inevitably results in reduced productivity (Bauer et al., 2016). The diminished productivity, in turn, often renders HRC-capable applications like collaborative screwing, illustrated in Figure 1, unattractive in industrial settings due to the combination of high investment costs and lower production efficiency.

However, precise prediction of human movements could contribute to anticipating safety requirements in HRC even before the actual movement occurs. This allows for adjustments to the robot’s movement speed, consequently enhancing production efficiency. To avoid such limitations like constant (slow) robot velocity, hybrid approaches like extending BMI with HAR can be used where the robot adapts to its human operator’s state. For maximal efficiency valuation of such approaches should be done in suitable research environments with HRC-capable and industry-oriented production system setups such as COssembly described by Christ et al. (2023). This procedure will be aimed as the next step for this hybrid approach.

## 7 Conclusion

In this article, we have reviewed the recent techniques and applications of BMI and HAR systems for decoding the human state in HRC. Each of these methods has its own strengths but also weaknesses limiting its use in a real industrial environment with real humans. The available data shows that by integrating the complementary information from BMI and HAR signals, a hybrid system can possibly achieve a more accurate and robust decoding of the human state especially when used for predictive adaptation of robot behavior. We have also discussed the potential benefits and challenges of using such a hybrid system as opposed to HAR and BMI alone, as well as the future directions and open questions in this field. We conclude that the fusion of BMI and HAR is a promising idea that can enhance the effectiveness and naturalness of HRC.
